# A curated DNA barcode reference library for parasitoids of northern European cyclically outbreaking geometrid moths

**DOI:** 10.1002/ece3.9525

**Published:** 2022-11-18

**Authors:** Tommi Nyman, Saskia Wutke, Elina Koivisto, Tero Klemola, Mark R. Shaw, Tommi Andersson, Håkon Haraldseide, Snorre B. Hagen, Ryosuke Nakadai, Kai Ruohomäki

**Affiliations:** ^1^ Department of Ecosystems in the Barents Region, Svanhovd Research Station Norwegian Institute of Bioeconomy Research Svanvik Norway; ^2^ Department of Environmental and Biological Sciences University of Eastern Finland Joensuu Finland; ^3^ Department of Biology University of Turku Turku Finland; ^4^ National Museums of Scotland Edinburgh UK; ^5^ Kevo Subarctic Research Institute, Biodiversity Unit University of Turku Turku Finland; ^6^ Kopervik Norway; ^7^ Biodiversity Division National Institute for Environmental Studies Tsukuba Japan

**Keywords:** barcoding, insect outbreaks, metabarcoding, molecular identification, parasitoid, population regulation

## Abstract

Large areas of forests are annually damaged or destroyed by outbreaking insect pests. Understanding the factors that trigger and terminate such population eruptions has become crucially important, as plants, plant‐feeding insects, and their natural enemies may respond differentially to the ongoing changes in the global climate. In northernmost Europe, climate‐driven range expansions of the geometrid moths *Epirrita autumnata* and *Operophtera brumata* have resulted in overlapping and increasingly severe outbreaks. Delayed density‐dependent responses of parasitoids are a plausible explanation for the 10‐year population cycles of these moth species, but the impact of parasitoids on geometrid outbreak dynamics is unclear due to a lack of knowledge on the host ranges and prevalences of parasitoids attacking the moths in nature. To overcome these problems, we reviewed the literature on parasitism in the focal geometrid species in their outbreak range and then constructed a DNA barcode reference library for all relevant parasitoid species based on reared specimens and sequences obtained from public databases. The combined recorded parasitoid community of *E. autumnata* and *O. brumata* consists of 32 hymenopteran species, all of which can be reliably identified based on their barcode sequences. The curated barcode library presented here opens up new opportunities for estimating the abundance and community composition of parasitoids across populations and ecosystems based on mass barcoding and metabarcoding approaches. Such information can be used for elucidating the role of parasitoids in moth population control, possibly also for devising methods for reducing the extent, intensity, and duration of outbreaks.

## Graphical abstract

We present a curated DNA barcode database for all parasitoid species associated with two geometrid species (*Epirrita autumnata* and *Operophtera brumata*) undergoing cyclic population outbreaks in the subarctic mountain birch forests that form the treeline in northern Europe. Our vetted database of host–parasitoid records and parasitoid barcodes constitutes a resource that can be used for barcoding‐ and metabarcoding‐based research on the significance of parasitoids for moth population control. Our barcode library will also enable molecular‐genetic monitoring of climate‐driven latitudinal shifts in moth–parasitoid networks and will facilitate the design of measures aiming at reducing the frequency and extent of moth outbreaks in northern European treeline forests.

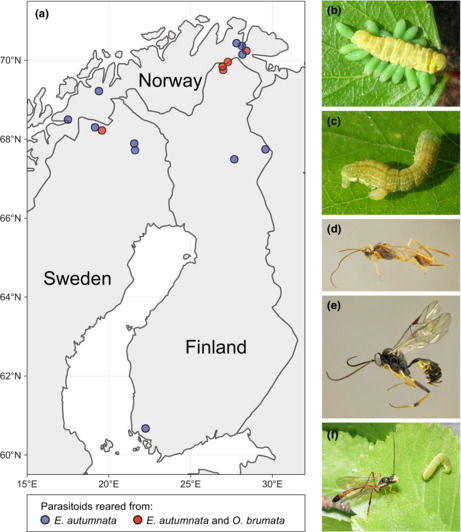

## 1 INTRODUCTION

Population outbreaks of plant‐feeding insects cause disturbances on ecosystems worldwide through destroying diversity and structure of plants that constitute the food and habitat of other animals and humans (Ayres & Lombardero, 2000; Eveleigh et al., 2007). Outbreak systems are highly heterogeneous and span a wide variety of different plant and insect species around the world (Ayres & Lombardero, 2000; Canelles et al., 2021). Because trophic (plant–insect–enemy) interactions and other potential regulatory mechanisms stabilizing insect population dynamics may be disrupted due to the rapidly changing global climate (Dyer et al., 2013; Pardikes et al., 2022; Romero et al., 2018; Thierry et al., 2019), understanding the abiotic and biotic drivers and consequences of insect outbreaks has become crucially important (Lehmann et al., 2020; Möller et al., 2017; Pureswaran et al., 2018). Thus far, however, lack of knowledge on biotic interactions between plant‐feeding insects and their natural enemies limits our understanding of the mechanisms underlying insect outbreaks.

Large‐scale population eruptions by geometrid moths in northern Europe constitute one of the most dramatic and thoroughly studied outbreak systems globally (Jepsen et al., 2016; Klemola et al., 2006; Tenow, 1972). Periodic outbreaks of the autumnal moth (*Epirrita autumnata* [Borkhausen]) are a naturally occurring phenomenon in the mountain birch (*Betula pubescens* var. *pumila* [L.] Govaerts) forests that form the northern and alpine treeline in Norway, Sweden, and Finland (Haukioja et al., 1988; Ruohomäki et al., 2000; Tenow & Bylund, 2000). The warming climate has, however, led to range expansion of another geometrid species, the winter moth (*Operophtera brumata* [L.]), into areas that historically experienced outbreaks of autumnal moth only (Ammunét et al., 2010; Jepsen et al., 2008, 2013; Vindstad et al., 2022). The now‐sympatric outbreak ranges of these species are likely to increase the frequency, intensity, and duration of forest defoliation in northern Europe (Neuvonen & Viiri, 2017; Vindstad, Jepsen, Ek, et al., 2019). Indeed, population peaks of winter moth often lag 1–2 years behind those of the autumnal moth (Klemola et al., 2008; Tenow et al., 2007), and severe defoliation exceeding 3 years from such combined outbreaks has resulted in forest dieback over large areas (Vindstad, Jepsen, Ek, et al., 2019). Even sublethal outbreaks fundamentally change local environments, as spillover herbivory and the sudden increase in light and nutrients cascade into shifts in communities of understory plants (Karlsen et al., 2013), root‐associated fungi (Saravesi et al., 2015), and soil invertebrates and microbes (Calderón‐Sanou et al., 2021). As shown by Heliasz et al. (2011), outbreaks also reduce sequestration of atmospheric carbon by mountain birch forests.

Due to the dramatic impacts that geometrid outbreaks have on subarctic treeline forest ecosystems, the factors that initiate and terminate outbreaks have been the focus of intensive research. Winter temperatures below −32°C reduce survival of overwintering moth eggs (Ammunét et al., 2012), and weather effects are discernible both in the spatial limits of local outbreaks (Hagen et al., 2007; Vindstad, Jepsen, Yoccoz, et al., 2019) and in the temporal outbreak dynamics (Karvinen, 2021). Repeated defoliation also lowers foliage quality for larvae (Kaitaniemi et al., 1999), and moth body size and fecundity are further suppressed as a result of resource depletion and feeding on non‐host plants in outbreak areas (Ammunét et al., 2010; Kaitaniemi et al., 1999; Klemola et al., 2008; Yang et al., 2008).

However, abiotic and resource‐related factors are likely to be either independent of moth density or to have an immediate (direct) impact, while the approximately 10‐year population cycles exhibited by both moth species rather point to the involvement of biotic factors operating in a delayed density‐dependent manner (Klemola et al., 2008; Ruohomäki et al., 2000). This suggests a role for top–down regulation by natural enemies such as predators and parasitoids (Berryman, 1996; Klemola et al., 2002), which theoretically also have the potential to synchronize population dynamics of different host species (Klemola et al., 2009; Raimondo et al., 2004). An effect of natural enemies was indeed demonstrated in an experiment by Klemola et al. (2010), who found that multi‐year exclusion of parasitoids led to higher densities of autumnal moth larvae in experimental cages.

Connecting parasitoid abundance to changes in moth densities has, however, proven difficult in natural settings (Hagen et al., 2010; Schott et al., 2010, 2012; Vindstad et al., 2010). A major challenge is lack of detailed knowledge on the host preferences and prevalences of particular parasitoid species in different areas and phases of moth population cycles (Klemola et al., 2008, 2014; Ruohomäki et al., 2000; Tenow, 1972; Vindstad, 2014). In this regard, the first methodological complication arises from difficulties in identifying parasitoids in multi‐species communities, which frequently include cryptic species that are difficult or impossible to separate based on morphological traits (Lue et al., 2021; Sigut et al., 2017). The parasitoid communities of autumnal and winter moths are species‐rich and ecologically diverse, involving species from at least 19 genera in five families (Klemola et al., 2007, 2014; Vindstad, 2014; Vindstad et al., 2010). However, identifications of species are known to be unreliable and naming schemes inconsistent across studies (Bylund, 1997; Klemola et al., 2007; Vindstad et al., 2011), which over time has led to perpetuation and amplification of errors (see discussion in Vindstad, 2014). Second, estimating species‐specific rates of parasitism in nature is difficult with traditional rearing methods, because such approaches are sensitive to the timing of sampling (Ruohomäki, 1994; Schott et al., 2010; Vindstad et al., 2011) and differential mortality during rearing (Ashfaq et al., 2004; Rott & Godfray, 2000; Sow et al., 2019).

Our goal here was to overcome these methodological challenges by developing molecular‐genetic resources enabling inference of parasitoid communities at both individual and ecosystem levels in the outbreak range of *E. autumnata* and *O. brumata* in northernmost Europe. Techniques based on DNA barcoding and genome‐level markers can effectively resolve complexes of cryptic or near‐cryptic species, but of the natural enemies of the focal moths, only the microgastrine genera *Protapanteles* and *Cotesia* have hitherto been investigated using molecular tools (Ruohomäki et al., 2013). Barcoding and metabarcoding approaches also allow obtaining data directly from herbivore larvae, without a need to rear hosts and parasitoids to adults (Kitson et al., 2019; Miller, Aguilera, et al., 2021; Miller, Polaszek, & Evans, 2021; Nakadai & Kawakita, 2017; Sow et al., 2019; Volf et al., 2017). Furthermore, when a reference barcode library for relevant parasitoids is available, their local community structures and abundances can be estimated based on large‐scale material obtained through, for example, Malaise trapping (Barsoum et al., 2019; DeWaard et al., 2019; Lue et al., 2021; Roslin et al., 2022).

**FIGURE 1:**
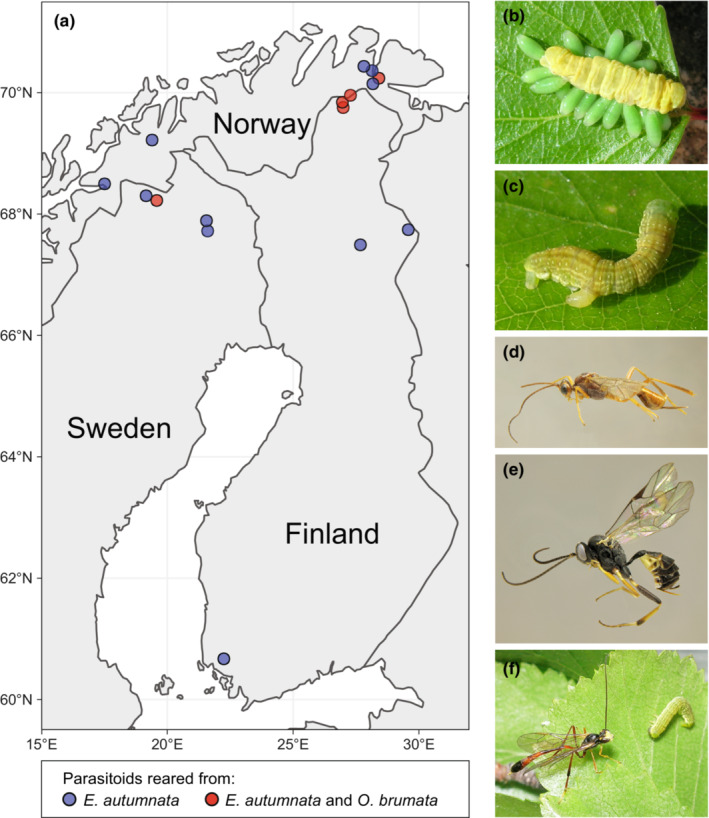
(a) Map of locations in Norway, Sweden, and Finland from which eggs, larvae, and/or pupae of *Epirrita autumnata* (blue dots) or both *E. autumnata* and *Operophtera brumata* (red dots) were collected for rearing of parasitoid reference specimens (see legend). Names and coordinates of the study sites are given in metadata S1 in Nyman et al. (2022). (b–f) examples of larval and adult parasitoids attacking the two focal outbreaking geometrid species: (b) gregarious ectoparasitic *Eulophus* sp. larvae feeding on a larva of *E. autumnata*, (c) an endoparasitic *Cotesia* or *Protapanteles* sp. larva exiting a larva of *E. autumnata*, (d) adult female of *Zele deceptor*, (e) adult female of *Phobocampe tempestiva*, and (f) female of *Agrypon flaveolatum* next to an *E. autumnata* larva. Photo credits: (b and c, f) Tero Klemola, (d and e) Anu Veijalainen (Zoological Museum, Univ. Turku, Finland).

To this aim, we reviewed the available literature on the natural enemies of *E. autumnata* and *O. brumata* in their outbreak range and constructed a reference DNA barcode library for the parasitoids based on material reared through a period of 15 years from eggs, larvae, and pupae collected from Norway, Finland, and Sweden (Figure 1). The barcode library presented here includes data from 132 reared parasitoid specimens and an additional 66 reference sequences obtained from public databases, together representing all of the 32 hymenopteran parasitoid species recorded to attack the focal moth species in their combined outbreak range. Our barcode library constitutes a resource that will in the future allow molecular identification of single parasitoid eggs, larvae, pupae, or adults, and will significantly expand possibilities for qualitative and quantitative molecular‐genetic assessment of parasitoid communities in populations of northern outbreaking moth species either from mass sampling of moth larvae or field trapping of adult parasitoids.

## 2 MATERIALS AND METHODS

### 2.1 Literature review of parasitoid communities

We assessed the completeness of our barcode library by reviewing the existing literature on parasitism on the focal geometrids in Finland, Norway, and Sweden, which encompass the main areas of their native outbreak range in the northern and mountainous parts of these countries (Jepsen et al., 2008; Klemola et al., 2006; Tenow, 1972). As far as we are aware, our review included all articles having direct observations of parasitoid species in the focal region (Table S1). Because literature records of host–parasitoid associations are often plagued by misidentifications of parasitoids, hosts, or both (Noyes, 1994; Shaw, 1994, 2017), limiting our focus to these countries ensured a higher reliability of recorded associations as well as a closer connection of recorded associations to outbreak dynamics of the focal geometrids. After this spatial delimitation, we inferred probable synonymies across parasitoid names in different sources and excluded records that apparently represented misidentifications. We included all published parasitoid species names in our curated community table (Table S1), but separately annotated records that in our view are unreliable or incorrect.

In addition to species composition, we compiled information on the ecology of the parasitoid species (Table S1). The associated parasitoids can be divided into distinct ecological guilds based on the developmental stage that they attack and/or kill and emerge from (eggs, larvae, prepupae, or pupae; Kenis et al., 2005; Klemola et al., 2007, 2014; Mills, 1994). The larval parasitoids can be further divided into those that develop inside or outside the larvae (endo‐ and ectoparasitoids, respectively) and into those that paralyze their host or allow it to continue development (idio‐ and koinobionts, respectively; Mills, 1994, 2009).

### 2.2 Sample collection, rearing, and morphological pre‐identification

We obtained parasitoid reference specimens from field‐collected larvae or from laboratory‐reared trap eggs, larvae, and pupae exposed in the field at 16 locations in Finland, Norway, and Sweden between 2004 and 2018 (Figure 1, metadata S1 in Nyman et al., 2022). Larvae were collected when the majority had molted to their penultimate (fourth) or ultimate (fifth) instar and reared on fresh birch foliage until pupation (Ruohomäki, 1994). Egg and pupal parasitoids were obtained by exposing sedentary life stages under field conditions in Hana, Norway (Klemola et al., 2014). Emerging specimens were stored in 1.5‐ml Eppendorf tubes in ethanol at +5°C or −20°C.

During the study years, over 3000 adult parasitoid wasps were reared from the moth hosts in focus. The specimens to be barcoded were selected based on morphological pre‐identifications done by K. Ruohomäki mostly to the species or genus level (metadata S1 in Nyman et al., 2022). To evaluate intraspecific sequence variation and to facilitate detection of potential cryptic taxa within presumed species or species‐groups, we barcoded 2–23 individuals of all those morphospecies for which multiple individuals were available (metadata S1 in Nyman et al., 2022).

### 2.3 DNA extraction, sequencing, and alignment

DNA was extracted using DNeasy Blood and Tissue Kits (Qiagen) following the manufacturer's protocol with slight modifications. All samples were lysed overnight in a thermomixer at 55°C between steps 2 and 3 in the manufacturer's protocol. At step 3, the lysis buffer (AL) was heated to 50°C before addition, and in step 7, the elution buffer (AE) was warmed to 70°C before addition. After pipetting buffer AE onto the spin column filter, the columns were incubated for up to 15 min at room temperature. The final elution was done twice into the same collection tube, leading to a total extract volume of 100 μl. Extract DNA concentrations were measured with a Qubit fluorometer using the Qubit 1X dsDNA HS Assay Kit (Invitrogen) following the manufacturer's protocol.

The standard barcode of the mitochondrial COI gene was PCR amplified with the universal primers LCO1490 and HCO2198 (Folmer et al., 1994). PCR reactions were carried out in volumes of 25 μl, including 2 μl template DNA, 0.5 μM of each primer, 1 U of *Taq* polymerase (Invitrogen), 0.2 μM of each dNTP, 1X Mg‐free PCR buffer, and 1.5 μM MgCl_2_. Thermal cycling conditions included initial denaturation at 94°C for 3 min, followed by 30 cycles of 94°C for 45 s, 50°C for 30 s, 72°C for 90 s, and a final extension at 72°C for 10 min. PCR products were checked through electrophoresis on 1.5% agarose gels stained with ethidium bromide. Whenever multiple bands were present in the gels, we performed a new PCR reaction with Q5 High‐Fidelity 2X Mastermix (New England BioLabs Inc.), in a total reaction volume of 25 μl, including 2 μl template DNA and 0.5 μM of each primer. These reactions were run with an initial denaturation at 98°C for 30 s, then 30 cycles of 98°C for 10 s, 50°C for 30 s, and 72°C for 30 s, followed by a final extension step at 72°C for 2 min.

Successfully amplified products were purified enzymatically from unincorporated nucleotides and primers before sequencing. For this, 15 μl of PCR product was mixed with 30 U Exonuclease I and 3 U FastAP Thermosensitive Alkaline Phosphatase (PCR cleanup prior to sequencing, Thermo Scientific), then incubated at 37°C for 15 min, followed by 85°C for 15 min to stop the reaction. The products were Sanger sequenced in both directions using the amplification primers at Macrogen Inc., The Netherlands.

Resultant sequences were edited and aligned using Geneious Prime 2020.1 software (Biomatters Ltd). Final sample sequences were aligned with each other using the MAFFT multiple sequence alignment algorithm (Katoh & Standley, 2013) implemented within Geneious. We constructed two different barcode sequence alignments for the subsequent analyses: (i) an alignment including only the 132 samples analyzed in this study; and (ii) an expanded alignment including a further 66 reference sequences retrieved from the GenBank and BOLD databases (metadata S1 and data S1 in Nyman et al., 2022). The reference barcode sequences for the latter dataset were selected to represent (i) identified reference individuals that constituted close hits for our own barcodes; (ii) identified specimens of species that our literature review indicated as parasitoids of the focal geometrids, but that were not obtained from our own rearings; and (iii) representative congeners of parasitoids that are known to parasitize the focal moth species. The last class also included five species that have been listed as parasitoids of the focal moth species, but that we consider doubtful or unlikely associates (Table S1).

### 2.4 Phylogeny reconstruction and species delimitation analyses

For the alignment of our own 132 barcode sequences, we first estimated a midpoint‐rooted Neighbor‐joining (NJ) based on Kimura 2‐parameter (K2P) distances in Mega X (Kumar et al., 2018) and used 1000 bootstrap resamplings of the data matrix to estimate clade support. Mega X was also used for calculating K2P distances among sequences within and between inferred species.

Next, we conducted a maximum‐likelihood (ML) analysis using RAxML v. 8 within Geneious Prime v 2020.1 (Stamatakis, 2014). In this case, we implemented a GTR + G substitution model but separated codon positions 1 and 2 from 3, following the two‐partition scheme suggested by PartitionFinder (Lanfear et al., 2017). Statistical support for groupings was evaluated with 1000 rapid bootstrap resamplings. A corresponding ML analysis was performed based on the 198‐sequence dataset containing the reference barcodes from GenBank and BOLD. Both ML trees were manually rooted between Platygastroidea+Chalcidoidea and the Ichneumonoidea following results of the phylogenomic analyses of Peters et al. (2017) and Branstetter et al. (2017).

We inferred limits among species by performing species delimitation analyses with the Bayesian implementation of the Poisson tree processes method of Zhang et al. (2013), which is available on the bPTP server (https://species.h‐its.org/ptp/). The method applies a single‐locus phylogenetic tree as input data to fit exponential distributions for the numbers of substitutions between within‐ and among‐species branching events on the tree and delimits species under the assumption that branches will on average be shorter within than among species. We used the ML tree estimated on the basis of our own barcode dataset as a guide tree and conducted MCMC sampling using a flat prior for all possible delimitations for 500,000 generations, with a burnin of 0.1 and the thinning parameter set to 100.

## 3 RESULTS

### 3.1 Literature review

Based on our review of 31 articles and own observations, 28 hymenopteran parasitoid species are recorded to attack *E. autumnata* and 17 species are recorded to attack *O. brumata* in northern Fennoscandia. Of these, 13 occur on both moth species, so their recorded collective parasitoid community consists of 32 hymenopteran species belonging to the families Platygastridae, Encyrtidae, Eulophidae, Braconidae, and Ichneumonidae (Table S1). We excluded a further nine names as probable synonyms, misidentifications, or rearing contaminants (see below). All of the hymenopteran species considered to represent real associates of the focal moth species are present in our barcode dataset, which also includes all of the likely but as yet unconfirmed associates (Table S1). Of the 32 species, 22 were found in our own reared material. The hymenopteran parasitoid community is dominated by larval and larval–prepupal parasitoids, but contains species representing attack on all immature stages of the focal moths (Table S1). Besides hymenopterans, *Lypha dubia* (Diptera: Tachinidae) infrequently attacks both moth species in the study area (K. Ruohomäki, personal observation); public barcode sequences for *L. dubia* species are available in BOLD (Table S1).

### 3.2 Barcode data and trees

We obtained barcode sequences for 132 reared parasitoid specimens. The full dataset including also representative reference sequences from GenBank and BOLD was composed of 198 sequences. Both alignments were 658 bp in length, but species‐specific barcodes were either 640, 643, 652, or 658 bp long, with differences being due to internal deletions of whole codon triplets (data S1 in Nyman et al., 2022). The shortest barcodes were found in two *Telenomus* sequences downloaded from GenBank.

The NJ and ML trees based on our own dataset differed in deep internal structure, but grouped the barcodes into corresponding clusters with 1–14 specimens in each (Figure S1–S3, data S2 in Nyman et al., 2022). The composition of these shallow clusters was identical across the trees, despite the fact that the backbone structure of the NJ tree is in clear conflict with the hymenopteran overall phylogeny (e.g., polyphyletic Chalcidoidea+Platygastroidea and Braconidae).

The ML solution of the bPTP species delimitation analysis based on the ML tree favored splitting the clusters into 22 species, with strong Bayesian support (>0.9) for many delimitations (Figure S2). However, the exact placement of some species limits remained uncertain. Surprisingly, many of the low Bayesian support values for alternative delimitations were found at or near the base of very tight barcode clusters, which were as such clearly distinct on the tree (e.g., *Telenomus* sp. 2, *Zele deceptor*, *Cratichneumon viator*, and *Phobocampe tempestiva*). When defining species based on the delimitation analysis, the mean within‐species K2P distance for species with more than one individual was 0.003 (range of mean = 0–0.012, s.e.m. = 0.0009), while the full range of intraspecific inter‐individual K2P distances was from 0 to 0.027. Mean K2P distances among individuals belonging to different sister species ranged from 0.025 to 0.261, and the mean interspecific distance among all species was 0.260 (range = 0.025–0.414, s.e.m. = 0.005).

**FIGURE 2:**
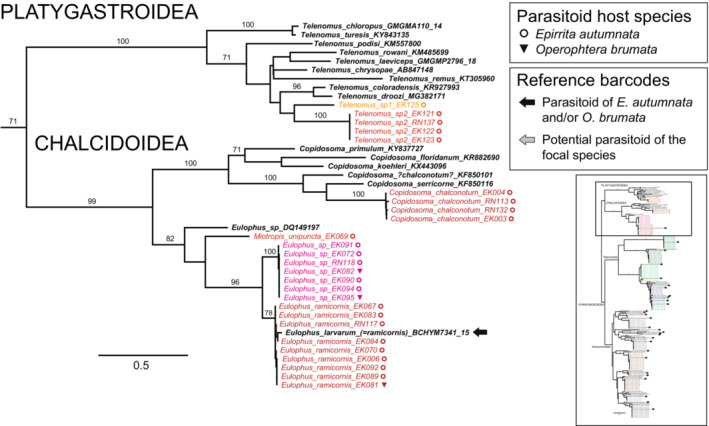
The Platygastroidea + Chalcidoidea clade of the full ML tree based on COI barcodes. The location of the clade in the full ML barcode tree (Figure S3) is indicated by the square in the inset figure. Specimens reared from *Epirrita autumnata* and *Operophtera brumata* are in colored fonts that correspond to different inferred species, reference specimens from GenBank and BOLD are in bold black font. Moth host species are indicated by symbols, and reference barcodes of species known or suspected to attack the focal moth species are indicated by arrows after names (see legends). Numbers above branches are bootstrap proportions (only values >70% shown).

**FIGURE 3:**
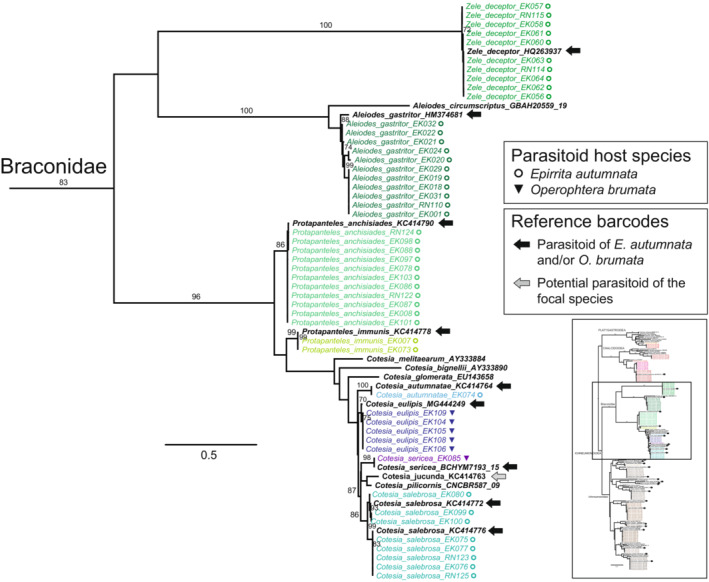
The Braconidae clade of the full ML tree based on COI barcodes. The location of the clade in the full ML barcode tree (Figure S3) is indicated by the square in the inset figure. Specimens reared from *Epirrita autumnata* and *Operophtera brumata* are in colored fonts that correspond to different inferred species, reference specimens from GenBank and BOLD are in bold black font. Moth host species are indicated by symbols, and reference barcodes of species known or suspected to attack the focal moth species are indicated by arrows after names (see legends). Numbers above branches are bootstrap proportions (only values >70% shown).

**FIGURE 4:**
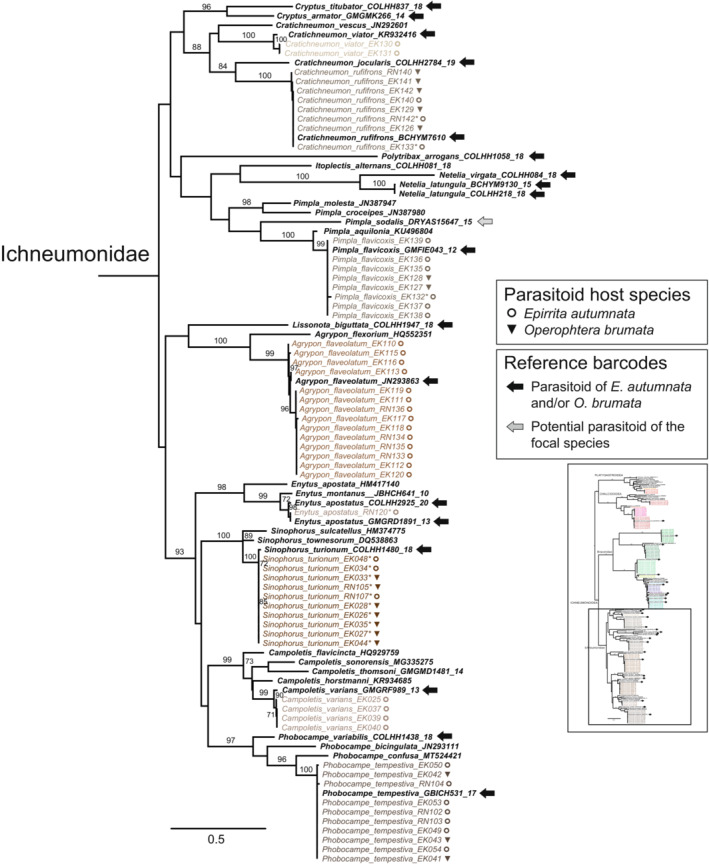
The Ichneumonidae clade of the full ML tree based on COI barcodes. The location of the clade in the full ML barcode tree (Figure S3) is indicated by the square in the inset figure. Specimens reared from *Epirrita autumnata* and *Operophtera brumata* are in colored fonts that correspond to different inferred species, reference specimens from GenBank and BOLD are in bold black font. Asterisks denote specimens for which the morphological preidentification was corrected based on comparisons to reference barcodes. Moth host species are indicated by symbols, and reference barcodes of species known or suspected to attack the focal moth species are indicated by arrows after names (see legends). Numbers above branches are bootstrap proportions (only values >70% shown).

The superfamily‐ and family‐level structure of the full 198‐sequence ML tree (Figure S3, data S2 in Nyman et al., 2022) corresponded with well‐established phylogenetic relationships among the main parasitoid taxa, although bootstrap support was low for most deep branching events. Inclusion of closely matching reference sequences from public databases confirmed our morphological identifications and allowed identification of most of the remaining taxa. Well‐separated single sequences or clearly delimited barcode clusters of associated parasitoids were present for two species in the Platygastroidea and four in the Chalcidoidea (Figure 2); three of these could not be identified to species level based on morphology or reference sequences obtained from barcode libraries. Within the Braconidae (Figure 3), barcodes of nine known or likely associate species were generally well‐defined in relation to each other, but maximum intraspecific K2P distances were comparatively long within the *Aleiodes gastritor* (0.022) and *Cotesia salebrosa* (0.014) clusters, and mean distances among individuals across species pairs were short within the *Cotesia* clade (0.025–0.039). All 17 known or likely parasitoids in the Ichneumonidae were similarly well‐separated and identifiable based on their barcode sequences (Figure 4). Within‐species divergences in Ichneumonidae were very low, with the exception of the *Agrypon flaveolatum* cluster, which contained a relatively deep split (maximum K2P distance 0.027).

## 4 DISCUSSION

DNA barcode reference libraries constitute a central resource for ecological research based on molecular‐genetic approaches (Morinière et al., 2019; Wirta et al., 2016). However, the extreme diversity of insects means that even the most comprehensive global or regional barcode libraries will at best contain only a subset of all species present in nature (Ratnasingham & Hebert, 2007; Roslin et al., 2022), and large‐scale databases also frequently include barcodes of misidentified individuals (Meiklejohn et al., 2019). Curated taxon‐ or system‐specific barcode libraries are therefore in many cases necessary for targeting relevant research questions and hypotheses (Lee et al., 2019; Lue et al., 2021; Nisole et al., 2020; Toro‐Delgado et al., 2022). Here, by drawing on the combined expertise of taxonomists, ecologists, and geneticists, we constructed a comprehensive DNA barcode library for parasitoids that attack the immature stages of the geometrid moths *E. autumnata* and *O. brumata* in their main outbreak range in northern Europe. A large body of literature exists on parasitism in these ecologically central moths, but, like for most other plant‐feeding insect groups, inferences have been hampered by misidentifications and inconsistent nomenclature (Klemola et al., 2007; Ruohomäki et al., 2013; Vindstad, 2014). By relying on vetted literature records and reared parasitoid specimens, we ensured that the associations are correct, and well‐documented reference barcodes for the few remaining species could be obtained from public databases. Our results show that all relevant parasitoids can be confidently identified to species level based on barcode sequences, but also reveal the presence of several new associates and putative cryptic species within the natural enemy community. Below, we first discuss the results and remaining taxonomic issues within each parasitoid superfamily and then outline ways for utilizing our barcode library in ecological and applied research on the drivers of population cycles in northern outbreaking geometrid moths.

### 4.1 Platygastroidea and Chalcidoidea

The hyperdiverse superfamilies Platygastroidea and Chalcidoidea globally contain thousands of species (Aguiar et al., 2013; Noyes, 2022; Rasplus et al., 2020) that, due to their typically minute size, are particularly challenging for morphological identification. Within Platygastridae, our barcodes revealed the existence of two species of *Telenomus* egg parasitoids separated by a K2P distance of 0.144. Neither of these matched reference sequences in GenBank or BOLD, but one or other of the species evidently represents “*Telenomus* cf. *laeviceps*,” which has previously been listed as an associate of *E. autumnata* (Ammunét et al., 2012; Klemola et al., 2009, 2014). Further work is required to pinpoint the exact taxonomic status of the two species found in our dataset, but we note that they are unlikely to represent the true *T. laeviceps* (reference GMGMP2796‐18), which is very distant from our barcode clusters on the ML phylogeny (Figure 2). In contrast to the inference of Barloggio (2018), the true *T. laeviceps* may therefore be associated exclusively with noctuid moths. Estimating rates of egg parasitism in the focal moths has proven challenging during years with low moth population densities, but attack rates are known to be high during and after outbreaks, making egg parasitoids likely candidates for population control in the outbreak range (Klemola et al., 2014).

A different situation is present in the Encyrtidae, in which our samples identified by an experienced specialist (Veli Vikberg) as *Copidosoma chalconotum* are likely to represent the correct name. Our barcodes produced a 99% hit to an unpublished *C. chalconotum* sequence from Norway on BOLD, so we consider the “*C. chalconotum*” GenBank reference sequence KF850101 (= BOLD GBAH8995‐14) to originate from a misidentification (Figure 2). The reference specimen was collected in China from an unnamed host species (Yu et al., 2014). *C. chalconotum* is a generalist attacking many moth families (Noyes, 2022), but is thought to be predominantly associated with geometrids in the subfamily Larentiinae (Guerrieri & Noyes, 2005; Yu et al., 2014), to which both of our focal moth species belong. *C. chalconotum* has been listed as an infrequent polyembryonic egg–larval or egg–prepupal endoparasitoid of *E. autumnata* in Finland (Teder et al., 2000) and Norway (Klemola et al., 2014), but also in the Alps (Kenis et al., 2005).

The Eulophidae are represented in our study by three well‐separated barcode clusters (Figure 2). Previously, only *Eulophus ramicornis* (listed as *E. larvarum* in earlier studies; see revision by Graham, 1988) has been known as a common gregarious larval ectoparasitoid of the focal moth species in northern Fennoscandia (Table S1). “*E. larvarum*” auctt (now *E. ramicornis*) is considered a wide generalist, but it is also the only chalcidoid parasitoid listed for *E. autumnata* in the Universal Chalcidoidea Database of Noyes (2022). Notably, the same database lists 12 further chalcidoid associates for *O. brumata* from outside our focal region. Despite being a widespread and common genus with over 70 described species, *Eulophus* is poorly represented in public databases, with few species‐level reference sequences available (BOLD Systems, 2022). Therefore, pinpointing the identity of our unidentified *Eulophus* sp. cluster will require further work. The cluster is 97.9% identical with unidentified Canadian Eulophidae barcodes in BOLD, but is clearly divergent from the real *E. ramicornis* (mean interspecific K2P distance = 0.123), indicating that the two groups likely represent distinct species (Figure 2). In a blind test, representative sibling vouchers of our barcoded individuals were identified as *E. ramicornis* by an experienced specialist (Richard Askew), so morphological differences between the species are evidently small or nonexistent. Our *Miotropis unipuncta* record (Figure 2) is based on a single individual and is therefore a possible rearing contaminant requiring further validation. *M. unipuncta* is generally considered a specialist parasitoid of *Coleophora* and other microlepidopterans (Noyes, 2022). However, the species is morphologically very variable and may represent a complex of multiple species (R. Askew, personal communication).

### 4.2 Ichneumonoidea: Braconidae

The braconid wasp community associated with the focal moths is composed of nine confirmed or likely species, many of which are common larval or larval–prepupal endoparasitoids (Table S1). Within the family, *Zele deceptor*, *Protapanteles anchisiades*, *P. immunis*, and *Aleiodes gastritor* formed distinct barcode clusters that also match publicly available reference sequences (Figure 3). However, the *A. gastritor* barcode cluster contains a substantial amount of heterogeneity, with a maximum within‐cluster K2P distance of 0.022. “*A. gastritor*” is a frequent parasitoid of many arboreal geometrid host species, but the name most likely encompasses a complex of several species with differing host preferences and overlapping intra‐ and interspecific variation in morphological traits and barcode sequences (M. R. Shaw, personal observation). All of our specimens originated from *E. autumnata*, which is also the only host listed for *A. gastritor* in previous studies on moth parasitism in our focal region (Table S1). However, *O. brumata* is attacked by a morphologically close but most likely different representative of the *A. gastritor* complex in the United Kingdom (M. R. Shaw, personal observation).

*Cotesia* is the taxonomically most complex genus in our focal host–parasitoid system (Figure 3). The genus is widespread and comprises over 300 species that are often very difficult to identify morphologically (Fernandez‐Triana et al., 2020). In the focal community, Ruohomäki et al. (2013) showed that barcodes can be used for separating *C. salebrosa* and *C. autumnatae*, and for distinguishing both from *C. jucunda*, which is known to parasitize *O. brumata* in Britain (M. R. Shaw, personal observation). The name “*C. jucunda*” is used in many of the studies included in our review (Table S1), but neither our results nor those of Ruohomäki et al. (2013) point toward an association of *C. jucunda* with outbreaking geometrids in northern Fennoscandia.

Our results support the notions of Klemola et al. (2012) and Ruohomäki et al. (2013) that *Cotesia* species exhibit differing preferences with regard to the two focal geometrid species. Of the four species observed here, only *C. salebrosa* has been listed as an associate of both *O. brumata* and *E. autumnata* (Table S1), even though all of our specimens were from the latter. *C. autumnatae* is apparently a strict specialist on *E. autumnata*, but sample sizes remain small due to the apparent rarity and southern distribution of the species (Ruohomäki et al., 2013). Of our specimens reared from *O. brumata*, five individuals pre‐identified as *C. eulipis* by M. R. Shaw formed a tight cluster with a *C. eulipis* reference barcode from Canada (MG444249). However, specimen EK085, likewise from *O. brumata* but lacking a species‐level pre‐identification, grouped with *C. sericea* reference BCHYM7193‐15 from the Czech Republic. As *C. sericea* has not previously been reported from Fennoscandia (Fernandez‐Triana et al., 2020), we reexamined 96 *Cotesia* specimens reared from *O. brumata* in two locations in Norway and Finland and found 51 *C. sericea* individuals in material collected through 2008–2010 (det. M. R. Shaw). Nixon (1974) listed the species (as *Apanteles praepotens*) as a regular parasitoid of *O. brumata* in the United Kingdom (also M. R. Shaw, personal observation), so it is likely that *C. sericea* is a recent addition to the parasitoid fauna of northern Europe that has gone unnoticed until now. Overall, while our results indicate that all *Cotesia* species associated with *E. autumnata* and *O. brumata* are separable based on COI barcodes (Figure 3), the relatively short interspecific distances mean that the question should be revisited in the future using larger sample sizes and combined population‐genomic and barcode data.

### 4.3 Ichneumonoidea: Ichneumonidae

The ichneumonid wasp community was found to be composed of 17 species that are larval, larval–prepupal, larval–pupal, or pupal parasitoids (Table S1). In our barcode tree, all ichneumonid species are well separated from each other, have low within‐cluster distances, and match publicly available reference sequences (Figure 4). The only exception to this general pattern is the deep split within the *Agrypon flaveolatum* barcode cluster. Our bPTP analysis placed the species limit at the base of the cluster with high support (Figure S2), but the maximum within‐cluster K2P distance is 0.027, which is close to the traditional yet arbitrary “species limit” of 2% sequence divergence applied in many barcoding studies (Hubert & Hanner, 2015). As pointed out by Vindstad (2014), the presence of two cryptic species cannot be excluded: Vindstad et al. (2010, 2013) found *A. flaveolatum* to parasitize both *E. autumnata* and *O. brumata* in several sites in the vicinity of Tromsø, Norway, while in a study by Klemola et al. (2009) from northernmost Finland, the species was absent from *O. brumata* larvae, and females refused to oviposit in this host in laboratory experiments despite high rates of attack on *E. autumnata*. The gap is not readily explained by hosts (all of our barcodes originated from individuals reared from *E. autumnata*) or geography (both clusters included samples from southern and northern Finland). Based on this, we consider the barcode cluster to represent a single species.

For the Ichneumonidae, past literature records contained a high number of cases that we consider unreliable (Table S1). For example, previous studies have listed at least five different species names within *Phobocampe*, but three of these represent apparent misidentifications or synonyms. *Phobocampe* species are morphologically very variable and available keys (e.g., Sedivý, 2004) can be considered unreliable. Furthermore, while we consider the pupal parasitoid *Pimpla sodalis* a likely associate of at least *E. autumnata*, the original record is based on indirect inference by Jussila and Nuorteva (1968): along with another ichneumonid, *Cryptus armator*, the species became very abundant through a population outbreak–collapse cycle of *E. autumnata* in Finnish Lapland in 1965–66, after having been absent in preceding years with “normal” moth densities.

*Enytus apostatus* is a new record for the parasitoid community of *E. autumnata* (Table S1). We only observed a single specimen and, as a broad generalist (Shaw et al., 2016), *E. apostatus* is likely to be neither common on the focal geometrids nor particularly relevant for their population dynamics. Nevertheless, the observation provides an illuminating example of the complexities of inferring parasitoid communities. Specimen RN120 was pre‐identified as “*Sinophorus* sp.,” but the barcode sequence confidently clustered with *E. apostatus* references from Norway and Germany (Figure 4). However, the BOLD database also includes more than a hundred predominantly North American barcodes under the name “*Enytus apostata*,” all of which are very distant from sequences of our specimen RN120 as well as available reference sequences of *E. apostatus* and the related *E. montanus* (Figure 4). Voucher photographs of “*E. apostata*” on BOLD do not seem to represent *Enytus*, and European sequences belonging to the same barcode index number (BIN) are identical to two Norwegian barcodes of *Hyposoter brischkei* (COLHH1404‐18 and COLHH1406‐18). Therefore, we consider the “*E. apostata*” barcode BIN in BOLD to represent a case in which the barcode of an originally misidentified *H. brischkei* specimen has been used to repeatedly (mis)label subsequent sequences added into the database.

### 4.4 Future prospects

Our curated DNA barcode reference library for the parasitoids of *E. autumnata* and *O. brumata* opens up attractive opportunities for elucidating the role of parasitoids in the eruptive population dynamics of geometrid moths in northern Europe. Rates of parasitism in insect herbivores are often very high, and delayed density‐dependent responses of parasitoids have frequently been implicated as a driver of host population cycles (Klemola et al., 2010, 2014; Münster‐Swendsen & Berryman, 2005; Mutanen et al., 2020; Myers, 2018; Turchin et al., 2003). However, inferences on whether—or which—parasitoids control outbreaking moth populations have been hampered by difficulties in species identification, inconsistent nomenclature, and differential rearing mortality (Vindstad, 2014). While the absolute and relative prevalences of particular parasitoid species vary through time and space (Ruohomäki, 1994; Teder et al., 2000; Tenow, 1972; Vindstad et al., 2010), we estimate that the 32 species included in our barcode library are responsible for nearly all of the total parasitoid‐inflicted mortality in the two moth species in the focal outbreak region. Importantly, our linking of barcodes to taxonomic names enables connecting genetic identifications to previously accumulated information on parasitoid ecology and life‐history traits (Table S1). This connection will allow tests of the relevance of species‐level biological traits, including host stage attacked and diet breadth, for parasitoid abundance and ecological impact.

The reference library presented here enables research implementing barcoding or metabarcoding approaches at the level of individuals, populations, and ecosystems. At the individual level, field‐collected parasitoid eggs, larvae, pupae, and adults can be readily identified based on barcode sequences, and rates of parasitism by externally inconspicuous endoparasitoids can be estimated using metabarcoding of DNA extracted from single host larvae (cf. Kitson et al., 2019; Miller, Polaszek, & Evans, 2021). For the latter type of studies, the availability of a parasitoid barcode reference library facilitates in silico validation of mini‐barcode amplification primers, as well as design of blocking oligomers suppressing simultaneous amplification of host DNA (Nakadai & Kawakita, 2017). At the population and ecosystem levels, parasitoid community composition can be estimated through metabarcoding of bulk Malaise trap catches (cf. Kirse et al., 2022; Sire et al., 2022). With appropriate trapping designs, parasitoid community structures can then be contrasted with spatial variation and multi‐year trajectories in host densities. What is more, the increasing output quality and decreasing cost of long‐read sequencing technologies means that truly quantitative community‐level mass barcoding approaches have become feasible (Hebert et al., 2018; Srivathsan et al., 2021). However, we note that such community‐level analyses based on both metabarcoding and mass barcoding methods would benefit from construction of more comprehensive barcode libraries for *all* parasitoids found in subarctic mountain birch forests, to ensure that potentially indistinguishable closely related parasitoid species that attack other insect species do not confound ecological inferences.

Like other arctic and subarctic ecosystems, the mountain birch forests of northern Europe are subject to rapid climatically induced changes (Pureswaran et al., 2018; Rees et al., 2020; Skre et al., 2017). Manifestations of these changes are already seen as shifts in the distributions of the focal moth species (Ammunét et al., 2010; Jepsen et al., 2013) and the extent of their outbreaks (Jepsen et al., 2008; Vindstad et al., 2022). With a warmer climate, additional geometrid birch defoliators are entering the region, potentially with cumulative impacts on subarctic treeline forests (Jepsen et al., 2011). However, parallel changes in parasitoid communities, host–parasitoid associations, and parasite‐mediated indirect interactions among moth species are expected, but are difficult to document (Kankaanpää et al., 2020; Vindstad et al., 2013). A substantial “reservoir” of additional geometrid moth parasitoids is known to exist further south in Europe, where moth population eruptions are less dramatic (Elkinton et al., 2021; Früh, 2014; Kenis et al., 2005; Noyes, 2022; Tikkanen et al., 1998; Vindstad et al., 2013; Wylie, 1960). Our curated moth parasitoid DNA barcode library integrating taxonomic, ecological, and molecular data therefore constitutes a reference point for monitoring changes in moth–parasitoid networks and their effects on moth population dynamics. As shown by the findings of Elkinton et al. (2021), a deeper understanding of the factors driving moth population dynamics may eventually provide tools for reducing the frequency and severity of geometrid outbreaks also in the treeline forests of northern Europe.

## AUTHOR CONTRIBUTIONS

**Tommi Nyman:** Conceptualization (lead); formal analysis (equal); funding acquisition (equal); investigation (equal); visualization (lead); writing – original draft (lead). **Saskia Wutke:** Conceptualization (lead); data curation (lead); formal analysis (lead); supervision (equal); writing – original draft (equal). **Elina Koivisto:** Data curation (equal); formal analysis (supporting); funding acquisition (equal); writing – original draft (supporting). **Tero Klemola:** Conceptualization (equal); funding acquisition (equal); investigation (equal); resources (equal); writing – review and editing (equal). **Mark R. Shaw:** Data curation (equal); investigation (supporting); resources (supporting); validation (lead); writing – review and editing (supporting). **Tommi Andersson:** Resources (supporting). **Håkon Haraldseide:** Resources (supporting); validation (supporting). **Snorre B. Hagen:** Conceptualization (supporting); writing – review and editing (supporting). **Ryosuke Nakadai:** Data curation (supporting); formal analysis (supporting); writing – review and editing (equal). **Kai Ruohomäki:** Conceptualization (equal); funding acquisition (equal); resources (lead); writing – review and editing (equal).

## Supporting information


Figures S1–S3

Table S1

## Data Availability

All new barcode sequences generated in this study have been deposited in GenBank (accession numbers LC735846–LC735977). The full 198‐barcode alignment including also publicly available reference sequences, the phylogenetic trees generated in this study, full collection data of our reared specimens, and GenBank/BOLD accession numbers of reference sequences included in the full analyses have been deposited in the Dryad Repository (Nyman et al., 2022; https://doi.org/10.5061/dryad.d51c5b067). Genomic DNA extracts of the analyzed specimens have been deposited in the Biobank of the NIBIO Svanhovd Research Station. Benefits from this research accrue from presenting a publicly available curated parasitoid DNA barcode database that can be used for basic and applied research on moth outbreaks in northern Europe and elsewhere. The work was made in compliance with national laws in Norway, Sweden, and Finland.
