# Supplementary material for: A curated DNA barcode reference library for parasitoids of northern European cyclically outbreaking geometrid moths
Source: Ecol Evol. 2022 Nov 18;12(11):e9525. doi: 10.1002/ece3.9525 (PMC9674473; doi:10.1002/ece3.9525)
Supplement: Supplementary file 1 — Figures S1–S3 [file ECE3-12-e9525-s001.pdf]

Ecology and Evolution
Open Access

Supporting Information for:

A curated DNA barcode reference library for parasitoids of northern European cyclically outbreaking geometrid moths

Tommi Nyman, Saskia Wutke, Elina Koivisto, Tero Klemola, Mark R. Shaw, Tommi Andersson, Håkon Haraldseide, Snorre B. Hagen, Ryosuke Nakadai, Kai Ruohomäki

Table S1. Parasitoids attacking *E. autumnata* and *O. brumata* eggs, larvae, prepupae, or pupae according to literature sources and the results presented here, with information on the ecological traits of the species. (.xlsx file)

Fig. S1. (A) NJ tree constructed on the basis of K2P distances among the 132 DNA barcode sequences produced in this study. (B) ML tree estimated based on the same dataset. Main parasitoid families and superfamilies are indicated next to branches, colors of specimen names correspond to different inferred parasitoid species, and moth host species are indicated by symbols after parasitoid names (see legend). In both trees, numbers above branches are bootstrap proportions (only values >70% shown).

Fig. S2. ML solution from the bPTP species delimitation analysis, with Bayesian support values shown above branches.

Fig. S3. Full ML tree constructed on the basis of 132 COI barcode sequences produced in this study and 66 reference sequences from GenBank and BOLD. Specimens reared from *E. autumnata* and *O. brumata* are in colored fonts that correspond to different inferred species, reference specimens from public databases are in bold black font. Moth host species are indicated by symbols (see legend), and reference barcodes of species known or suspected to attack the focal moth species are indicated by arrows after names (see legend). Numbers above branches are bootstrap proportions (only values >70% shown). Squares surrounding the three main clades of the tree indicate the main parasitoid groups shown separately in Figs. 2–4.

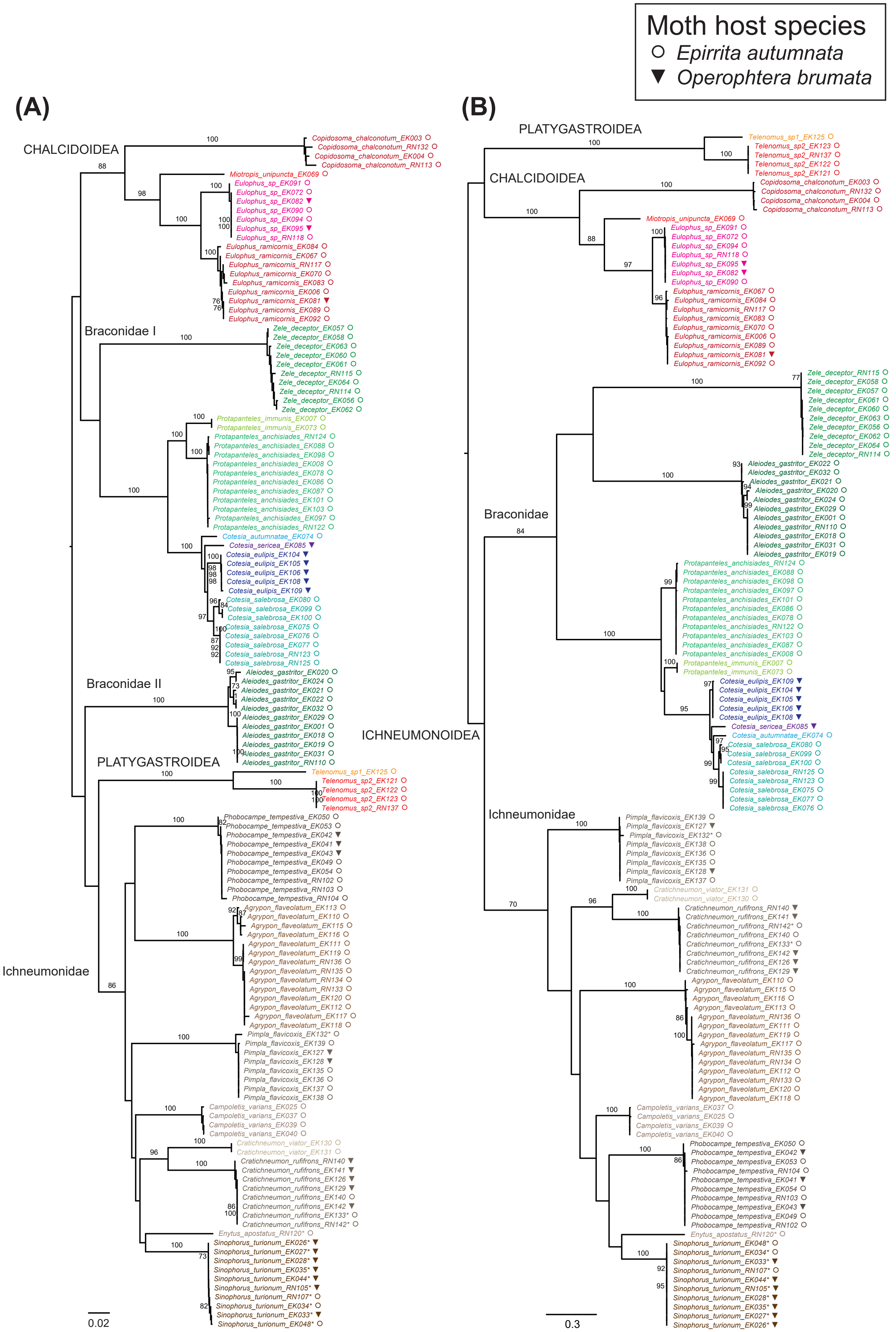

Supporting Information Fig. S1

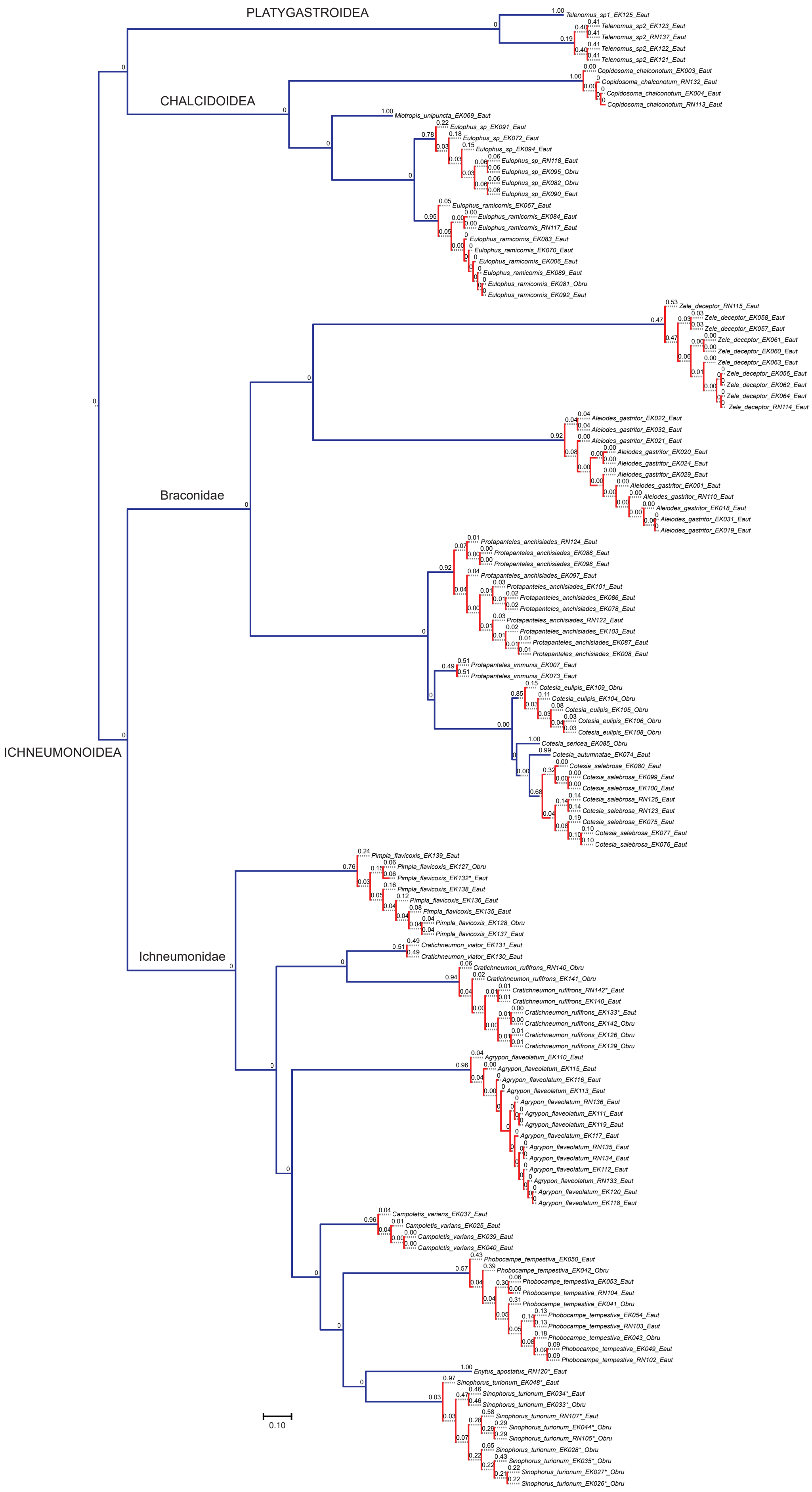

Supporting Information Fig. S2

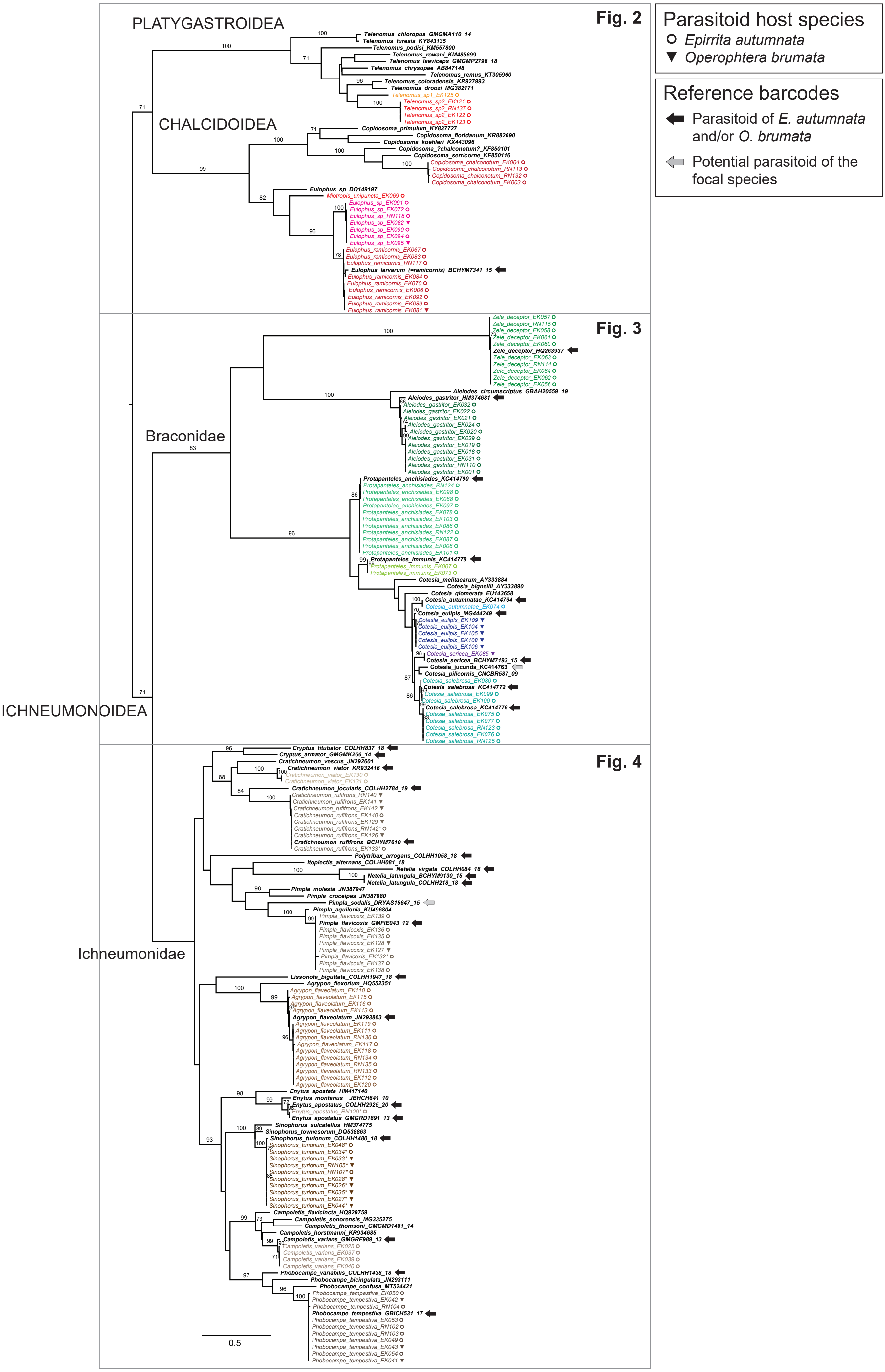

Supporting Information Fig. S3
